# Structural elements and theoretical logic of the ought-to L2 self: an inductive theory-building analysis

**DOI:** 10.3389/fpsyg.2026.1862853

**Published:** 2026-07-03

**Authors:** Zhiyu Liu, Meixi Dong, Lei Zhang, Huizi Li, Hanrui Zhang, Xianzhi Wang

**Affiliations:** 1 College of Foreign Languages, North China University of Science and Technology, Tangshan, China; 2 School of Foreign Studies, University of Science and Technology Beijing, Beijing, China; 3 Department of International Cooperation, North China University of Science and Technology, Tangshan, China

**Keywords:** inductive analysis, internalization, L2 motivational self system, ought-to L2 self, second language acquisition, structural elements, theoretical logic

## Abstract

**Introduction:**

The ought-to L2 self, a key component of the L2 Motivational Self System, has predominantly been conceptualized as a single-dimensional variable in quantitative research, leaving its internal structure and operational logic under-theorized. This study addresses this gap by deconstructing the ought-to L2 self and constructing a theoretical model of its structural elements and theoretical logic.

**Methods:**

Adopting an inductive theory-building analysis informed by grounded theory coding procedures, this study conducted a systematic analysis of a diverse and topic-focused dataset that included academic monographs, peer-reviewed journal articles, and learners' discussions from online platforms.

**Results:**

Through iterative open, axial, and selective coding, the conventionally single-dimensional construct was decomposed into a tripartite model. Ten main categories were identified in the coding: career development motivation, academic development motivation, language capital motivation, motivation from significant others, social regulation pressure, internalization of obligation, anticipatory emotions activation, discrepancy perception, strategic learning engagement, and habituated practice. These categories cluster into three dimensions: external drivers, psychological processing, and behavioral responses, which collectively demonstrate the construct's inherently blended motivational nature.

**Discussion:**

This study further advances the exploration of the dimensionality of the ought-to L2 self, demonstrating that this construct possesses a blended motivational nature rather than a fixed prevention focus. Practically, it provides L2 educators with conceptual references that may inform possible pedagogical directions.

## 1 Introduction

Learning motivation is a key and widely researched construct in the field of second language acquisition (SLA). The L2 Motivational Self System (L2MSS), as a core framework for understanding foreign language learners' motivation, posits that learner behavior is driven by three fundamental components: ideal L2 self, ought-to L2 self, and L2 learning experience (Dörnyei, 2005, 2009; Dörnyei et al., 2014; Dörnyei and Ryan, 2015). Among them, the ought-to L2 self concerns the attributes that one believes one ought to possess to meet expectations and to avoid possible negative outcomes (Dörnyei, 2009). Empirical findings (Siridetkoon and Dewaele, 2018; Wu and Liu, 2023) have indicated that language learners with a robust ought-to L2 self are likely to obtain motivation to acquire the target language, primarily driven by a concern over disappointing important figures like their teachers. This highlights the preventive nature of the ought-to L2 self, in contrast to the promotion-focused ideal L2 self, which primarily embodies the learners' aspirations and positive desires (Dörnyei, 2005, 2009)

Nevertheless, the theoretical conceptualization of the ought-to L2 self is increasingly viewed as insufficiently elaborated. While the classic definition emphasizes a “prevention focus”, the drive to avoid failure or disappointing others (Dörnyei, 2005, 2009), scholars (Chen, 2012; Quinto and Castillo, 2016) argue that it may also operate with a “promotion focus”, reflecting aspirations for pursuing personal goals and external expectations. This conceptual tension has spurred scholarly attempts to expand its dimensionality, ranging from the introduction of the “anti-ought-to” self (Thompson and Vásquez, 2015), capturing resistance to external pressures, to the bifurcation into “ought-to/own” and “ought-to/others” (Papi et al., 2019; Teimouri, 2017). Although these refinements acknowledge the construct's multifaceted nature, they are insufficiently refined to construct an integrated psychological system that reflects the inherent complexity of psychological phenomena.

Current research on the ought-to L2 self has evolved along two primary investigative paths. First, its construct validity has been consistently corroborated within broader investigations of the L2MSS across varied cultural and educational contexts, particularly in Asian countries like China, Korea, and Indonesia (Khaleghizadeh et al., 2025; Subekti and Glory, 2022; You and Dörnyei, 2016). The second path is more prevalent and represents the current mainstream of research. Previous research has explored the relationship between the ought-to L2 self and various learner-internal and external factors, such as learner engagement (Lee and Lee, 2020), students' attitude to translanguaging (Yamagami, 2023), L2 learning experience (Huang et al., 2025), and growth language mindset (Jiang et al., 2024). These existing studies primarily incorporate the ought-to L2 self as a single-dimensional variable into analytical frameworks, focusing on its interrelation with learning motivation and behaviors, while seldom providing systematic theoretical examination of its internal composition. The current research orientation has left the fundamental problem unresolved regarding what structural elements constitute the “ought-to L2 self” and by what theoretical logic these elements form an integrated whole.

The objective of this study is to address this theoretical gap by re-conceptualizing the ought-to L2 self from a single-dimensional construct to a complex one with an internal structure. Departing from the hypothesis-testing nature of quantitative inquiries, this study adopts an inductive theory-building analysis of mixed data to facilitate a more granular, data-driven investigation into the construct's structure. Through clarifying the interrelationships among its structural elements, this study aims to elucidate the theoretical logic underlying the operation of this construct. It is important to note that while the analysis draws on systematic coding techniques, it does not claim to offer a fully grounded account of individual learner experience, but rather integrates both scholarly discourse and online posts from language learners. The findings endeavor to offer a more refined conceptual definition and a referential analytical framework for future research, equipping L2 educators and curriculum designers with conceptual references that may inform possible pedagogical directions.

## 2 Literature review

### 2.1 Ought-to L2 self

Dörnyei's (2009) L2MSS is a widely recognized framework for understanding L2 motivation (Toohey et al., 2025). Building upon Gardner (1985) socio-educational model and Markus and Nurius (1987) possible selves theory, the L2MSS conceptualizes motivation as the desire to reduce the discrepancy between one's actual self and future possible selves (Dörnyei and Ushioda, 2009). The framework comprises three key dimensions: the ideal L2 self, the ought-to L2 self, and the L2 learning experience (Dörnyei, 2005). Central to this study is the ought-to L2 self, which captures motivation driven by external expectations and the desire to avoid negative consequences, such as disappointing others or failing to meet social obligations (Dörnyei, 2005, 2009).

Although its role has been investigated across different contexts (Kim, 2009; Subekti and Glory, 2022; You and Dörnyei, 2016), the theoretical core of the ought-to L2 remains notably ambiguous. The classic definition of this construct emphasizes its “prevention focus”, which refers to the motivation to learn a second language in order to avoid disappointment, criticism, or failure (Dörnyei, 2009). This framing positions the learners primarily in a defensive stance, driven by the need to evade negative outcomes. However, subsequent research has revealed a more complex scenario. Scholars (Chen, 2012; Quinto and Castillo, 2016) argue that in collectivist cultures, fulfilling the expectations of significant others such as parents and teachers may carry positive emotional value and facilitate the attainment of social identity. In such contexts, meeting external expectations is not merely a duty but a pathway to social integration and personal affirmation. In this sense, the ought-to L2 self also includes certain features of a “promotion focus”, which is typically a characteristic of the ideal L2 self. The study by Siridetkoon and Dewaele (2018) also found that the fear of disappointing teachers (prevention focus) and the desire to meet family expectations (implying promotion focus) jointly influence second language learning behaviors. Thus, the learners' motivation may simultaneously be shaped by the urge to avoid loss and the aspiration to achieve relational harmony, creating an internally blended motivational force.

This conceptual tension between “prevention” and “promotion” orientations reflects a deeper theoretical issue. Existing research (Jiang et al., 2024; Huang et al., 2025; Wu and Liu, 2023) predominantly treats the ought-to L2 self as a single-dimensional and homogeneous construct. This oversimplified view obscures the intricate psychological reality learners experience. However, the very concept of “ought-to” may inherently encompass multiple and even potentially conflicting components. Henry and Liu (2024) have noted the fundamentally multifaceted nature of the ought-to L2 self in their critique of the L2MSS construct validity. These components include expectations from different social agents such as family, teachers, and peers, different perceptions of responsibility such as moral obligation or pragmatic avoidance, and different emotional motivations such as fear, guilt, or the desire for recognition. However, the existing literature lacks a systematic exploration of these potential internal dimensions and fails to clarify how these dimensions are interconnected and organized into a coherent structure of the ought-to L2 self. Therefore, this construct remains theoretically under-deconstructed, and the theoretical logic governing its internal operations urgently requires clarification.

### 2.2 Current research approaches to the ought-to L2 self

The current mainstream paradigm in empirical research on the ought-to L2 self imposes methodological constraints on the exploration of its internal structure. Existing studies (Huang et al., 2025; Lee and Lee, 2020; Wu and Liu, 2023) predominantly employ quantitative research approaches, such as structural equation modeling or regression analysis, treating the construct as a predefined variable measured holistically through scales. These studies then examine its relationships with antecedent variables (e.g., family influence, social atmosphere) and outcome variables (e.g., learning strategies, perseverance, anxiety, and academic performance). Such studies have undoubtedly accumulated a wealth of evidence demonstrating the predictive power of the ought-to L2 self as an aggregate variable.

However, the prevailing quantitative approach has its inherent limitations. First, reliance on the original or adapted items of the ought-to L2 self in established scales, such as the scale developed by Taguchi et al. (2009), essentially presupposes that it is a single-dimensional construct, thereby failing to detect dimensions that may exist within the data but are not covered by existing theories. Second, although relevant quantitative analyses can only reveal co-variation among variables, they lack the capacity to elucidate the constituent elements of the construct and their underlying theoretical logic. For example, while Papi (2010) found that the ought-to L2 self is positively correlated with second-language anxiety, the study could not determine whether “fear of disappointing parents” or “concern about failing to meet professional requirements” was the main driver of this anxiety. A similar limitation is evident in the study by Jiang et al. (2024). Even when introducing variables such as “growth language mindset”, Jiang et al. (2024) still treat the ought-to L2 self as a single-dimensional construct, without revealing which specific dimensions within it interact with the growth language mindset.

To address the limitations of viewing the ought-to L2 self as a single dimension, scholarly efforts have evolved toward more conceptualizations. Initially, (Thompson and Vásquez 2015) offered a critical revision by introducing the “anti-ought-to” dimension, capturing motivation rooted in resistance to external expectations. This perspective is particularly crucial in contexts where language learning is a low priority (Toohey et al., 2025), explaining why learners may defy dominant societal attitudes (Yousefi and Mahmoodi, 2022) and reject prescriptive notions of effort allocation (Toohey et al., 2025). Building upon this trend toward multiplicity, subsequent research (Papi et al., 2019; Teimouri, 2017) proposed a more structured bifurcation, dividing the construct into the ought-to L2 self/own and the ought-to L2 self/others. Papi et al. (2019) provide preliminary evidence supporting the validity of their proposed model and suggested that L2 motivation research should adopt a broader scope, encompassing a wider range of motives with diverse regulatory orientations. This distinction delineates between self-imposed obligations and pressures from external sources (Huang et al., 2025), with empirical studies substantiating its construct validity and contextual relevance within specific domains such as EFL (Wang and Zeng, 2025).

Despite these advancements in broadening the dimensionality of the construct, both the critical and the dichotomous models are insufficiently refined to constitute an integrated psychological system capable of reflecting the inherent complexity of psychological phenomena. Therefore, a more granular, data-driven investigation is required to unveil the underlying structure of the ought-to L2 self, which aligns with Papi et al. (2019) suggestion for further studies to disentangle the multi-dimensional nature of this construct by incorporating a wider spectrum of motivations rooted in both internal and external regulatory pressures. Accordingly, the present study adopts an inductive theory-building analysis of mixed textual data to deconstruct the ought-to L2 self and propose a theoretical model of its structural elements and underlying logic.

## 3 Methodology

### 3.1 Research design

This study conducts an inductive theory-building analysis of mixed textual data, drawing on the systematic coding procedures of procedural grounded theory (Strauss and Corbin, 1998) to facilitate the development of a conceptual model from systematically analyzed data. The choice is justified by the conceptual status of the ought-to L2 self, which remains underdeveloped and insufficiently deconstructed in L2 motivation research. Unlike quantitative designs that rely on predefined constructs and hypotheses, this inductive approach enables theoretical insights to emerge inductively through constant comparison and iterative abstraction, making it well suited to complex, context-bound psychological phenomena (Charmaz, 2006). Crucially, while this study employs grounded theory's coding techniques (open, axial, and selective coding) to ensure analytic rigor, it does not claim to offer a fully grounded account of individual learner experience; rather, it integrates scholarly discourse with learner-generated narratives to map the construct's latent complexity. This design allows the internal structure and operational logic of the ought-to L2 self to be mapped from the data, moving beyond its conventional treatment as a single-dimensional variable.

### 3.2 Data collection

The research data were collected from secondary textual sources, providing access to a wide array of established arguments, empirical findings, and informal discourse pertinent to the ought-to L2 self. The textual corpus was compiled from three primary categories: related chapters from academic monographs, peer-reviewed journal articles, and online posts from social platforms. This study acknowledges that while secondary textual materials are recognized as legitimate inputs for inductive analysis (Charmaz, 2006; Glaser and Strauss, 2017), the use of such mixed data necessitates careful handling to avoid conflating established theories with emergent patterns. This design aligns with the study's aim to reconstruct the socially and discursively constituted internal structure of the ought-to L2 self, rather than document idiosyncratic experiences in a single context. Therefore, these data sources combine scholarly rigor for credible theory-building with contextual richness drawn from real-world debates and learner experiences pertaining to the ought-to L2 self.

To ensure the transparency of the data corpus, this study systematically documented the sourcing, screening, and selection procedures for each data category. Regarding the monographs, two foundational ones were purposefully selected based on their seminal contribution to the conceptualization of the L2 Motivational Self System: *Motivation, Language Identity and the L2 Self* (2009) and *Motivational Dynamics in Language Learning* (2014). Related chapters within these volumes that offered in-depth discussion regarding the ought-to L2 self were identified for coding. For the peer-reviewed journal articles, a systematic search was conducted in the Scopus database to ensure a focused and manageable corpus. The search query utilized the term “ought-to L2 self”. The initial retrieval yielded 84 articles. To construct a targeted corpus, two inclusion criteria were applied: (1) a primary focus on the ought-to L2 self rather than merely mentioning it in passing, and (2) publication within the time-frame of 2020 to 2025, ensuring the relevance of contemporary scholarship. Following the application of these criteria, a purposive sampling strategy was employed to select 20 articles from the filtered pool, ensuring both diversity in learning contexts and a balanced distribution across publication years. Detailed information is presented in the Supplementary Table 2.

To prevent potential methodological ambiguity regarding the use of prior scholarship, a specific data screening protocol was applied to academic texts (including both monographs and journal articles). To maintain the inductive integrity of the analysis and prevent the model from being overly constrained by existing theoretical frameworks, highly theoretical definitions (e.g., “the ought-to L2 self concerns the attributes that one believes one ought to possess to meet expectations and to avoid possible negative outcomes.”) were excluded to prevent preconceived frameworks. Instead, the analysis focused on general descriptive statements, such as “Studying English for a promotion is related to one's ought-to L2 self.” These statements were analyzed alongside learner discourses to inductively generate categories.

Regarding the online discussions, the sampling strategy involved purposeful selection from three major social media platforms (TikTok, Douyin, and Xiaohongshu). To avoid omitting relevant experiential accounts, the initial retrieval used a broad search term: “second language learning” and its Chinese equivalent “二语学习” to locate posts (videos or texts) related to L2 learning contexts. The sampling procedure was documented in three sequential steps: post identification, comment extraction, and final inclusion screening. From the comment threads under these posts, units were then screened according to explicit inclusion criteria aligned with the ought-to L2 self.

Specifically, each comment unit was retained only if it met two conditions: (a) referred to personal second language learning experiences; and (b) expressed or implied externally imposed obligations, expectations, or responsibilities to learn a second language. The first author systematically reviewed all retrieved comments against the two inclusion criteria, which were applied consistently throughout the screening process. As a result, both coverage and relevance were ensured, with all 96 finally sampled discussion posts fully satisfying the stated conditions. Table 1 provides an overview of the data corpus, detailing the composition of sources across different categories and the number of coded excerpts extracted from each.

**Table 1:** Composition of the data corpus.

Source type	Title/Platform	Year/Period	Language	Country/Learning context	No. of coded excerpts
Monographs	*Motivation, Language Identity and the L2 Self*	2009	English	Multiple countries like Iran, Japan, Korea, and China	28
	*Motivational Dynamics in Language Learning*	2014	English	Multiple countries like China, UK, Iran, and Germany	24
Articles	Examining the Effects of the L2 Learning Experience on the Ideal L2 Self and Ought-to L2 Self in a Japanese University Context; other 19 articles (refer to the Supplementary Table 2)	2020–2025	English	Multiple contexts: China, Japan, Korea, Thailand, Ethiopia, Vietnam, Iran, Australia, Saudi Arabia, Chile	34
Online posts	TikTok	2020–2025	English	Multiple countries like Israel, Japan, UK, and Brazil	23
	Douyin	2020–2025	Chinese	China	38
	Xiaohongshu	2020–2025	Chinese	China	35

### 3.3 Data analysis

The data were organized and analyzed systematically using Microsoft Excel spreadsheets to support the inductive coding process, enabling the categorization, constant comparison, and theoretical development of the data through an iterative approach. Through a process of line-by-line analysis and constant comparison, open coding of 182 excerpts drawn from three distinct source types yielded 66 preliminary concepts. These concepts were subsequently refined and aggregated into 27 higher-order categories. For instance, instances of “mandatory language threshold for immigration” were consolidated under the broader category of “overseas development needs”. Axial coding then established logical relationships among categories through paradigmatic modeling, yielding 10 main categories that constitute the structural elements of the ought-to L2 self. Finally, selective coding integrated all main categories into a coherent theoretical narrative anchored in the core category: structural elements and theoretical logic of the ought-to L2 self. This integration produced a tripartite model linking external drivers, psychological processing, and behavioral responses.

To demonstrate the bottom-up analytical procedure, the construction of three main categories (career development motivation, internalization of obligation, and discrepancy perception) is presented as substantive examples corresponding to three respective dimensions in the theoretical model. With respect to the categorization process of career development motivation, open coding allowed the researchers to decompose diverse excerpts into seven discrete preliminary concepts, such as “mandatory language threshold for immigration” and “occupational promotion needs”. These excerpts ranged from explicit policy regulations to general empirical observations. For example, explicit policy regulations included the statement (e.g., “For applying for Canada's Federal Skilled Worker Immigration, language scores are a hard requirement”). In addition, general empirical observations included the statement (e.g., “Studying English for a promotion is related to one's ought-to L2 self in the same way that it is in China”). The seven preliminary concepts were then logically grouped and abstracted into three distinct categories: career promotion needs, overseas development needs, and job-hunting motivation. Axial coding then progressed from aggregation to defining theoretical dimensions and clarifying category relationships. Through constant comparison, these interrelated categories were synthesized into the main category of career development motivation.

Concerning the categorization process of internalization of obligation, open coding dissected diverse data excerpts to yield four preliminary concepts: cognitive internalization of external requirements, self-attribution of responsibility, self-acceptance of instrumental value, and self-requirements derived from social comparison. Subsequently, axial coding consolidated these concepts into two categorical dimensions: cognitive reconstruction and value recognition. Ultimately, through constant comparative analysis, these interconnected categories were abstracted into the overarching main category of internalization of obligation. With regard to the categorization process of discrepancy perception, researchers extracted four preliminary concepts from raw data: identification of competence deficiencies, phased self-evaluation, quantitative analysis of competence gap, and temporal perception of discrepancy. These concepts were then assembled *via* axial coding into two main categories: current status monitoring and standard comparison. Through constant comparison, these two interrelated categories crystallized into the main category of discrepancy perception. This demonstrates that such categorization processes were not theory-driven but emerged inductively from constant data comparison.

### 3.4 Research reliability and validity

To guarantee the reliability and validity of the research findings, several verification procedures were implemented. First, a rigorous coding process was conducted in collaboration with two specialists specializing in language education to validate the data. This approach was instrumental in mitigating researchers' biases and ensuring consistency in data analysis. Second, throughout the study, a systematic practice of keeping reflexive memos was adopted, aimed at documenting both preexisting and emergent information for constructing theory. Such methodological rigor not only allowed for a more profound interpretation of the data but also bolstered the validity of the results. Third, the two researchers independently coded the data, and inter-coder reliability was assessed using the simple agreement rate formula, resulting in a score of 91.66%, calculated as (number of units coded identically by both researchers / total units coded)^*^100%. To address the remaining 8.34% of discrepancies, this study adopted a structured consensus-coding procedure. This process required both researchers to jointly review the original data context and refer back to the evolving analytical memos. Disagreements were resolved by either reaching a consensus on a single code or refining the code definitions to better capture data nuances. Accordingly, this iterative resolution process served not only to ensure coding precision but also acted as a critical mechanism for refining the conceptual model.

### 3.5 Ethical statements

This study utilized posts exclusively sourced from the publicly accessible sections of TikTok, Douyin, and Xiaohongshu, ensuring that no private accounts, direct messages, or restricted content were accessed. The research design strictly adhered to the terms of service and privacy policies of all three platforms. Specifically, TikTok authorizes data sharing with independent scholars (TikTok Technology Limited, 2025), while Douyin (Section 2.2.3) permits the use of anonymized data for academic work (Beijing Douyin Technology Co. Ltd, 2026). Similarly, the privacy policy of Xiaohongshu (Section I, Article 4, Clause 5) permits the processing of voluntarily shared public information in accordance with Chinese privacy regulations (Xingyin Information Technology Co., Ltd., 2025).

The study aligns with platform permissions, local laws, and institutional rules. Under these guidelines, research using public social media data without direct human contact is exempt from formal ethical review and informed consent requirements. Therefore, this study appropriately determined that no ethical approval was necessary. To protect participant anonymity, all user-generated content was de-identified prior to analysis by removing usernames, avatars, and account identifiers, while avoiding any alteration to the original meaning. Additionally, translation from Chinese to English was performed by bilingual researchers and verified through back-translation checks to ensure semantic accuracy and consistency.

## 4 Results

### 4.1 Open coding

In the initial phase of open coding, researchers adopted an open and inductive stance toward the raw data, engaging in constant comparison to conceptualize and categorize emergent ideas (Glaser and Strauss, 2017). This iterative analytical process continued until theoretical saturation was reached, whereby no new concepts emerged from the data. For the focal construct of the ought-to L2 self, saturation was reached after coding all the 52 excerpts from the book chapters, 31st excerpt from journal articles, and the 90th excerpt from online posts, when no new categories arose. Once unrelated information was removed from the raw data, a total of 182 original excerpts were retained. These were then subjected to detailed word-by-word labeling and coding, which led to the development of 66 preliminary concepts. These concepts were subsequently organized into 27 categories, based on shared thematic similarities.

Taking the categorization process of “overseas development needs” category as an example, one original sentence (“Migrants will be forced to learn English to A Level standard if they want to move to Britain.”) was extracted from the data. The coding process began with line-by-line labeling of this excerpt, generating initial tags such as “immigration precondition” and “English proficiency standard”. Through constant comparison with similar data (e.g., “For applying for Canada's Federal Skilled Worker Immigration, language scores are a hard requirement.”), these fragmented tags were abstracted into the preliminary concept of “mandatory language threshold for immigration”. This preliminary concept, alongside the other two concepts of “language requirement for long-term residence” and “necessity for overseas survival”, were grouped together after identifying their shared core attribute: language prerequisites driven by external demands in overseas contexts. Accordingly, these three preliminary concepts finally fell under the category of “overseas development needs”.

Due to limited space, a subset of the open coding results (six categories) is presented in Table 2. The remaining 21 categories derived from the open coding are listed in Table 3. The complete coding results are available in the Supplementary Table 1.

**Table 2:** A subset of findings from the open coding.

Category	Preliminary concept	Representative original excerpt
Career promotion needs	Occupational promotion needs	Studying English for a promotion is related to one's ought-to L2 self in the same way that it is in China.
	Requirement for promotion and salary raise	The majority of Chinese people living in mainland China aspire to gain promotion at work in order to secure a higher salary that would be used to support family members. This reason is associated with their ought-to L2 self.
Overseas development needs	Mandatory language threshold for immigration	Migrants will be forced to learn English to A Level standard if they want to move to Britain. If you come to this country, you must learn our language and play your part.
	Language requirement for long-term residence	The message will be very clear... if you want to come to Quebec for more than three years... you have to speak French.
	Necessity for overseas survival	我不得不把英语学好, 然后用它谋生, 然后能够出国。 (Translation: I have to learn English well, use it to make a living, and be able to go abroad.)
Job-hunting motivation	Mandatory language threshold for job-hunting	Without knowing the German language, the chances of getting a job are very less. German language at B1-B2 level has become the minimum requirement for most jobs.
	Competitive edge in job-hunting	I perceive English as a great advantage in obtaining a good job after I go back to China, so I paid attention to accumulating terminology and knowledge...
Academic advancement needs	Necessity for further academic studies	Mastery of English vocabulary and grammar greatly contributes to my success at university entrance exam.
	Mandatory requirement for postgraduate entrance examination	考研初试考得好的话, 复试要考英语 (Translation: If you perform well in the preliminary exam for the postgraduate entrance exam, you will still be required to take an English test as part of the re-examination at the interview stage.)
	Necessity for studying abroad	不想考研了转战出国, 也要学英语。(Translation: If you give up on taking the postgraduate entrance exam and switch to preparing for overseas study, you'll still have to study English.)
Academic assessment pressure	Motivation for passing language tests	I am learning English in order to pass the IELTS exam.
	Pressure from course assessment results	Knowing classroom performance accounts for 40% of our overall grade, I suddenly realized that in English-only classes I must be very conscientious about every word out of my mouth!
Educational system requirements	Mandatory requirement for compulsory courses	For example, you might be studying a language because it's a mandatory school subject.
	Language requirement of universities	If you are learning a language for practical purposes–for example, to meet a college language requirement–then you are guided by an instrumental orientation.
	Rigid requirement for graduation	I have to learn English because without passing the English course I cannot graduate.

**Table 3:** Main categories formed by the axial coding.

Main category	Category	Category description
Career development motivation	Career promotion needs	The perceived obligation to meet workplace language proficiency standards for career advancement
	Overseas development needs	The duty to acquire foreign language skills to fulfill overseas work/study requirement
	Job-hunting motivation	The need to meet employers' language competency expectations during job searches, rooted in labor market norms
Academic development motivation	Academic advancement needs	The obligation to achieve academic milestones (e.g., graduation, further study abroad) through L2 proficiency
	Academic assessment pressure	The pressure to meet institutional assessment criteria (e.g., exams, thesis) that mandate L2 competence, reflecting academic norms
	Educational system requirements	The duty to comply with curriculum-mandated L2 learning objectives set by educational authorities
Language capital motivation	Information acquisition medium	The recognition of L2 (English) as an obligatory tool to access global information, driven by societal demands for international engagement
	Global function of English	The perceived obligation to master English to fulfill its global functions (e.g., international communication, academic collaboration, and professional engagement)
	Economic benefit motivation	The obligation to learn L2 for career-related economic rewards, driven by market-based expectations of language as capital
Motivation from significant others	Parental motivation	The duty to meet parents' expectations for L2 achievement, rooted in familial obligations and cultural values
	Peer influence	The competitive pressure to match or exceed peers' L2 proficiency levels or learning behaviors
	Teacher influence	The obligation to meet teachers' academic expectations for L2 learning (e.g., mastering specific linguistic skills, and achieving set proficiency benchmarks)
Social regulation pressure	Social evaluation pressure	The pressure arises from societal evaluation criteria (fueled by ingrained social beliefs, negative social judgment, and stereotype-driven expectations) that equate L2 competence with personal worth
	Social expectations	The duty to achieve community-wide L2 proficiency expectations (e.g., meeting required competencies for educated individuals, fulfilling student role responsibilities)
Internalization of obligation	Cognitive reconstruction	The process of reinterpreting external L2 obligations as personal responsibilities, shaping one's self-regulatory framework
	Value recognition	The internalization of L2′s instrumental value (e.g., access to cutting-edge information) and social comparison-driven self-demands (e.g., keeping up with peers' international exchange opportunities) as personal imperatives
Anticipatory emotions activation	Prevention focus	The activation of fear or concern about failing to meet L2 obligations, motivating precautionary learning behaviors
	Expected sense of efficacy	The belief in one's ability to fulfill L2 obligations (e.g., achieving expected proficiency through positive imagination of success or avoiding regret *via* psychological drive)
	L2 Learning anxiety	The anxiety arising from the obligation to meet external L2 standards, reflecting internalized pressure to avoid negative judgment
Discrepancy perception	Current status monitoring	The awareness of gaps between current L2 ability and externally imposed standards, triggering obligation -based action
	Standard comparison	The process of comparing one's L2 performance against normative benchmarks, fueling the sense of duty to close gaps
Strategic learning engagement	Target orientation	The orientation toward externally defined L2 goals (*via* targeted practice and time-focused investment)
	Meta-cognitive regulation	The strategic self-management of L2 learning (*via* method adjustment and resource utilization)
	Resource integration	The active combination of resources (*via* multi-channel input and socialized learning) to fulfill L2 obligations
Habituated practice	Behavioral automation	The automatic engagement in L2 learning behaviors (through fixed learning rituals and unconscious initiation)
	Learning pattern solidification	The stabilization of L2 learning routines (through stable learning rhythms and embedding into daily scenarios)
	Context-triggered behavior	The initiation of L2 learning behaviors in response to context-specific external triggers (e.g., environmental cues) rather than just exams or deadlines

### 4.2 Axial coding

Axial coding builds on open coding by identifying and establishing connections between categories to derive main categories, which are then systematically reintegrated according to their inherent logical relationships (Strauss and Corbin, 1990).

In this study, axial coding focused on uncovering the higher-order dimensions that unified seemingly disparate categories. For instance, during the analytical process, three distinct categories emerged: job-hunting motivation (reflecting the need to meet employers' competency expectations), career promotion needs (representing the obligation to meet workplace standards for advancement), and overseas development needs (stemming from requirements for immigration or long-term overseas survival). Although these originated from different contexts (labor market, internal promotion, and international mobility), constant comparison revealed a shared core logic: all were driven by external socioeconomic demands and future-oriented instrumental goals where English proficiency served as a mandatory prerequisite for achieving tangible benefits. Consequently, these three categories were integrated under the overarching main category of “career development motivation”. This process of grouping was replicated across the data to form the remaining nine main categories, ensuring that the final structure was derived inductively from the data rather than theoretically imposed. The resulting 10 main categories, along with their subordinated categories and detailed descriptions, are displayed in Table 3.

### 4.3 Selective coding

Selective coding is the process of identifying a core category that conceptually integrates and explains all the categories identified in the data, aiming to create a coherent and integrated theoretical model that accounts for the core phenomenon being studied (Strauss and Corbin, 1990). To encapsulate both the components and their operational mechanisms, this study adopts the “structural elements and theoretical logic of the ought-to L2 self” as the core category. In this context, theoretical logic is defined as a conceptual taxonomy of category relations derived inductively from the data, serving to unify the diverse elements into a single explanatory system.

**Figure 1:**
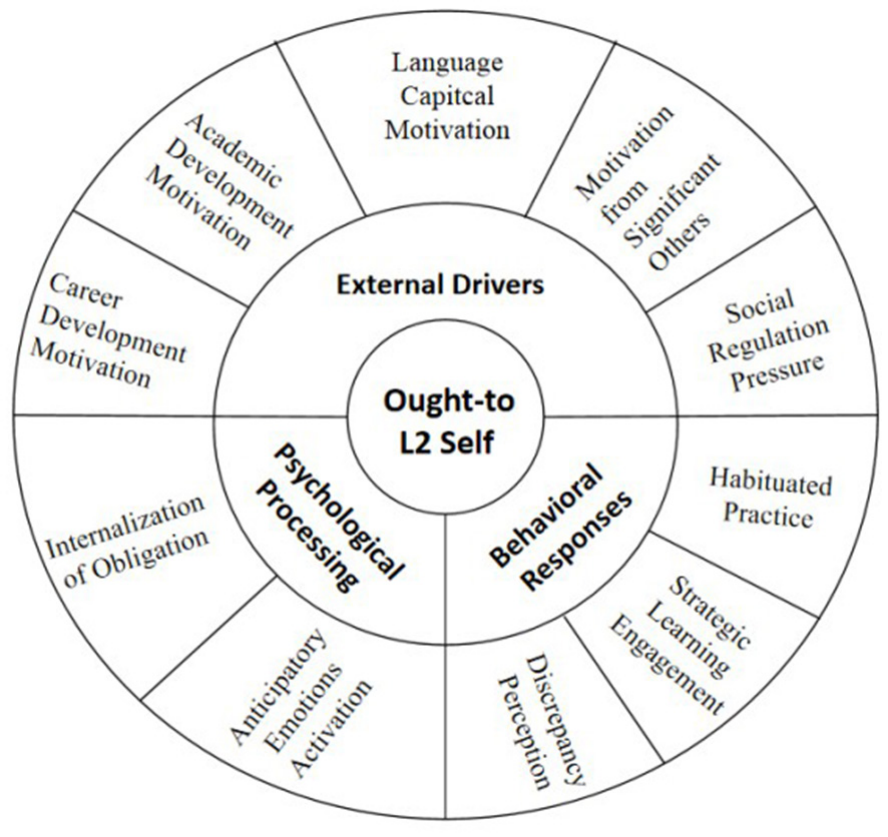
Structural elements and theoretical logic of the ought-to L2 self.

Through constant comparison, the 10 main categories were organized into three conceptually coherent clusters. The first cluster, external drivers, encompasses career development motivation, academic development motivation, language capital motivation, motivation from significant others, and social regulation pressure, capturing the diverse sources of obligation referenced across the corpus. The second cluster, psychological processing, comprises internalization of obligation and anticipatory emotions activation, reflecting learners' cognitive and affective responses to these external demands. The third cluster, behavioral responses, includes discrepancy perception, strategic learning engagement, and habituated practice, mapping onto observable learning behaviors and self-regulatory efforts. The relationships among these three clusters are characterized as conceptual alignment and data co-occurrence, rather than verified causal sequences. This model delineates a proposed conceptual structure linking the sources, processes, and responses associated with the ought-to L2 self. By decomposing the conventionally treated single-dimensional construct into distinct yet interrelated components, it clarifies the internal complexity of the ought-to L2 self. Collectively, these category structures and their conceptual alignments constitute the structural elements and theoretical logic of the ought-to L2 self presented in Figure 1.

### 4.4 Theoretical saturation

Theoretical saturation served as the guiding criterion for determining the adequacy of the sampling depth (Glaser and Strauss, 2017). This study adopted an iterative approach of theoretical saturation assessment consistent with the inductive analysis of mixed textual sources. Saturation was assessed throughout the coding process via the constant comparative method, where each newly collected datum was compared with existing codes and categories. During the analysis, no new categories emerged after coding all the 52 excerpts from the book chapters, 31st excerpt from journal articles, and the 90th excerpt from online posts. Analysis of remaining data sources yielded redundant information that fit within the established theoretical model. This iterative process of comparison and categorization confirmed that theoretical saturation had been reached, indicating that the data sufficiently supported the complexity of the emerging model without necessitating further sampling.

## 5 Discussion

Drawing on an inductive theory-building analysis of mixed textual data, this study constructs a tripartite conceptual model of the ought-to L2 self (Dörnyei, 2005, 2009; Higgins, 1987), delineating its structural elements and theoretical logic. The model delineates 10 specific elements clustered into three dimensions: external drivers, psychological processing, and behavioral responses. This model significantly extends the traditionally broad definition of the ought-to L2 self (Dörnyei, 2009; Taguchi et al., 2009) by disaggregating it into distinct internal components. This granularity not only refines the conceptual boundaries of the ought-to L2 self but also illuminates the heterogeneous nature of motivational forces in second language learning.

### 5.1 External drivers of the ought-to L2 self

The first dimension of the ought-to L2 self comprises external drivers, including career development motivation, academic development motivation, language capital motivation, motivation from significant others, and social regulation pressure. The components of career development motivation, which include career promotion needs, overseas development needs, and job-hunting motivation, underscore the role of L2 competence as a strategic asset for professional advancement. Empirically, Japanese learners associated English improvement with long-term career growth (Saito et al., 2017), while Chinese and Iranian learners viewed high proficiency as critical for competitive employment and international mobility (Taguchi et al., 2009), confirming that L2 skills are strategically internalized as professional capital. However, this motivation operates under distinct boundary conditions, presenting a competing psychological reality. Specifically, high-stakes career aspirations can induce severe language anxiety among career-focused students (Wang, 2025) or trigger learning burnout and disengagement during intensive job preparation (Safian et al., 2025). Thus, career development motivation is ambivalent, shifting from a strategic asset to a psychological burden depending on external pressure levels.

Academic development motivation, comprising academic advancement needs, academic assessment pressure, and educational system requirements, functions as a guiding framework that affects L2 learning within institutional contexts. Recent L2 motivation research (D'Orazzi, 2024) suggests that setting initial academic goals of deciding to learn a language functions as a key psychological driver for subsequent learning, shaping learners' commitment and strategic approaches to language acquisition. The presence of academic assessment pressure as a distinct category is particularly noteworthy, as it often manifests in the form of heightened anxiety during assessment tasks. Aladini et al. (2024) acknowledge this link between assessment demands and learner anxiety, identifying the provision of supportive assessment experiences as a crucial factor for fostering positive engagement and motivation. Therefore, only under deliberately supportive high-stakes testing conditions can learners' motivation be positively shaped, leading to improved performance and deeper learning engagement.

Under language capital motivation, the categories of information acquisition medium, global function of English, and economic benefit motivation highlight the symbolic and economic capital attached to L2 mastery. Grounded in Bourdieu's concept of cultural capital, recent scholarship posits that English, functioning as a global lingua franca, operates as a convertible asset that grants individuals access to informational flows, transnational networks, and economic resources (Bourdieu, 1991; Bourdieu and Passeron, 1977; Li, 2020). Beyond its cultural and symbolic value, proficiency in English constitutes a critical form of economic capital. It not only bolsters individuals' competitiveness within local and international labor markets but, critically, acts as a salary premium that facilitates access to high-wage employment and elite professional trajectories (Loyalka et al., 2025; Peltokorpi and Xie, 2025).

Motivation from significant others, including parental motivation, peer influence, and teacher influence, captures the relational dynamics that shape L2 motivation. A growing body of research (Guo et al., 2025; Pham, 2023; Solhi, 2024; Zhang, 2025) demonstrates how parents, peers and teachers function both individually and collectively to shape learners' L2 motivation. This working mechanism is not single-dimensional. According to self-determination theory (Ryan and Deci, 2000), when learners internalize these expectations from significant others autonomously, they act as facilitative drivers for persistence and engagement. Conversely, when these social pressures are perceived as controlling or excessive, the ought-to L2 self becomes a psychological burden. Under such boundary conditions, intense parental expectations or teacher demands can backfire, triggering foreign language anxiety, avoidance, and disengagement rather than productive motivation (Choi et al., 2019; Hasanzadeh et al., 2024). Thus, the motivational outcome of significant others depends heavily on whether their influence supports learner autonomy or imposes adverse emotional strain.

Social regulation pressure, consisting of social evaluation pressure and social expectations, addresses the normative dimension of L2 motivation. This construct aligns with Papi et al. (2019) notion of prevention-focused self-guides, which capture learners' sensitivity to social duties and the apprehension of potential failure. However, the motivational outcome of this pressure depends on whether the source of expectations is internalized. When learners accept social duties as personal obligations, this pressure acts as a facilitative driver. Under this condition, the perceived risk of failure promotes regulatory vigilance, prompting learners to sustain persistent investment in L2 learning and adopt vigilant strategies to avoid negative outcomes (Papi et al., 2019). On the contrary, when social regulation pressure operates as a purely external mandate, it undermines adaptive functioning. Excessive social evaluation and chronically heightened normative pressure deplete the learner's coping capacity, triggering defensive avoidance or psychological resistance (Papi and Khajavy, 2021; Teimouri, 2017).

### 5.2 Psychological processing of the ought-to L2 self

The second dimension of the ought-to L2 self pertains to psychological processing: internalization of obligation and anticipatory emotions activation. Internalization of obligation involves cognitive reconstruction, which redefines external L2 learning demands as personal responsibilities, and value recognition, which transforms L2-related benefits into personal goals. This psychological shift from external pressure to internal commitment is crucial, as it determines whether the learner operates under controlled regulation or moves toward more self-determined forms of motivation. Drawing on self-determination theory, Deci and Ryan (2000) argue that the internalization of extrinsic regulations, such as recognizing the value of L2 learning or integrating it with one's sense of self, facilitates more autonomous and sustained engagement. In the L2 context, this aligns with the process of “identified regulation” where learners accept the personal importance of language learning even if the initial impetus was external (Ryan and Deci, 2000).

Anticipatory emotions activation consists of prevention focus, expected sense of efficacy, and L2 learning anxiety, reflecting fear of failure, confidence in success, and the tension between external pressures and personal capabilities. According to regulatory focus theory (Higgins, 1997, 1998), individuals operating under a prevention focus are particularly sensitive to potential losses and failures, generating anticipatory anxiety when obligations remain unfulfilled. In second language learning, prevention-based anticipatory fear often reflects the ought-to L2 self, driving learners to meet external expectations and avoid failure (Dörnyei, 2009). In addition, expected sense of efficacy reflects Bandura (1997) self-efficacy, denoting a learner's pre-task conviction in their capability to succeed in upcoming L2 actions. This forward-looking appraisal determines emotional response: high expected efficacy generates anticipatory confidence and persistence, while low expected efficacy triggers anxiety and avoidance when confronting challenges (Bandura, 1997). Thus, expected sense of efficacy bridges self-perception and affect, dictating whether learners approach future L2 tasks with assurance or apprehension.

Recent empirical evidence highlights a dual-function relationship between L2 learning anxiety and the ought-to L2 self. On one hand, anxiety stemming from perceived obligations and external pressures can serve a facilitating role. By amplifying the perceived consequences of failure, this form of anxiety enhances self-regulatory vigilance, motivating learners to invest greater effort to meet external standards and avoid negative outcomes (Luo and Xiong, 2025; Welesilassie and Nikolov, 2025). On the other hand, high-intensity obligation-induced anxiety often exerts debilitating effects. When the emotional burden becomes excessive, it depletes cognitive resources, leading to avoidance behaviors, mental blockage, and diminished intended effort, thereby hindering proactive engagement with the L2 (Papi and Khajavy, 2023; Zhang and Lai, 2024).

### 5.3 Behavioral responses of the ought-to L2 self

The third dimension of the ought-to L2 self involves behavioral responses, including discrepancy perception, strategic learning engagement, and habituated practice. Discrepancy perception comprises current status monitoring, tracking the gap between actual L2 proficiency and external standards to trigger obligation, and standard comparison, which evaluates performance against norms to reinforce the duty to bridge that gap. Grounded in Higgins (1987) self-discrepancy theory, this perception of a proficiency gap represents the cognitive appraisal of the actual-ought discrepancy, wherein the learner's current L2 competence is evaluated against external standards or obligations. This misalignment constitutes a psychological state of “the presence of negative outcomes”, which activates prevention-focused strategies and agitation-related emotions (e.g., anxiety and guilt) to compel L2 learners to fulfill their perceived duty.

Strategic learning engagement entails target orientation toward external goals, meta-cognitive regulation of learning processes, and resource integration of multi-channel inputs to fulfill obligations. From the perspective of self-regulated learning theory (Zimmerman, 2000), this process reflects learners' proactive agency, wherein they strategically allocate time and effort to bridge self-appraised proficiency gaps. Unlike generalized learning behaviors, strategic learning engagement is characterized by a distinct instrumental rationality, with its orientation stemming directly from the need to respond to specific discrepancies. This intentional allocation of effort reflects a strategic approach to learning, where learners select, sequence, and monitor learning activities based on their self-appraised gaps and desired outcomes. Empirical studies (Cheng et al., 2025; Zimmerman, 2002) substantiate this view, demonstrating that learners who deploy such strategic behaviors, including goal setting, planning, and self-monitoring, exhibit higher achievement and are more effective in bridging the gap between actual performance and desired standards.

Habituated practice is characterized by behavioral automation (unconscious execution *via* fixed rituals), learning pattern solidification (the institutionalization of stable routines), and context-triggered behavior (actions initiated by situational stimuli rather than explicit goals). These features mark a qualitative shift in the underlying mechanism of motivation. The driving force behind the ought-to L2 self shifts from a state of conscious effort and persistence to one of spontaneous and natural engagement. Goal-oriented learning ceases to depend on continuous willpower and instead becomes ingrained as a routine aspect of everyday behavior. Indeed, this progression is consistent with research on habit formation, which suggests that repeated behaviors, when consistently paired with stable contextual cues, become automatized and require less conscious effort over time (Stojanovic et al., 2022; Wood and Neal, 2007). In second language learning, this means that strategies initially adopted through deliberate control can, through repetition, become habitual routines that support long-term maintenance of the ought-to L2 self.

## 6 Conclusion

Drawing on an inductive theory-building analysis of mixed textual data, this study refines the conceptualization of the ought-to L2 self, traditionally treated as a single-dimensional construct, by re-conceptualizing it as a multidimensional psychological system. Through systematic investigation of its structural elements and theoretical logic, the research deconstructs the construct into a tripartite model encompassing external drivers, psychological processing, and behavioral responses, which together comprise ten structural elements. This granular decomposition further advances the exploration of the dimensionality of the ought-to L2 self, demonstrating that this construct inherently possesses a blended motivational nature rather than a fixed prevention focus.

The proposed tripartite model may offer conceptual implications for L2 teaching and curriculum design. First, given that the ought-to L2 self is driven by diverse external sources, educators should implement differentiated motivation intervention. For instance, professional English courses can emphasize career development motivation, while compulsory education should mitigate excessive social evaluation pressure to reduce anxiety. Second, educators should prioritize facilitating obligation internalization. Rather than relying solely on external pressure, teachers should guide learners to recognize the instrumental value of L2 proficiency and provide supportive scaffolding to convert external demands into personal commitments. Third, the model points to the potential role of habituated practice in supporting sustained engagement. By helping learners establish stable learning routines and meta-cognitive strategies, instructional design may help shift the burden of learning from high-pressure and conscious effort toward automatic and sustainable behavior.

While this study provides a comprehensive deconstruction of the ought-to L2 self, several limitations warrant attention. First, this study draws on secondary textual sources, including academic literature and social media posts. While this design ensures broad data diversity and theoretical saturation, it limits access to the fine-grained and individualized processes that direct learner engagement data (e.g., interviews, classroom observations) could reveal. Future research should therefore integrate such primary data to test, validate, and further refine the proposed theoretical model. Second, although the model builds on data from both Chinese and Western contexts, it does not systematically examine how cultural backgrounds and learning stages may shape the ought-to L2 self, limiting the model's generalizability to underrepresented learner groups. Future studies could validate the model across specific cultural and proficiency subgroups, with comparative analyses between Eastern and Western learners and across learning stages to further refine its applicability.

## Data Availability

The original contributions presented in the study are included in the article/Supplementary material, further inquiries can be directed to the corresponding author.
